# Supplementary material for: Ancient environmental DNA reveals shifts in dominant mutualisms during the late Quaternary
Source: Nat Commun. 2018 Jan 10;9:139. doi: 10.1038/s41467-017-02421-3 (PMC5762924; doi:10.1038/s41467-017-02421-3)
Supplement: Supplementary file 1 — Supplementary Information [file 41467_2017_2421_MOESM1_ESM.pdf]

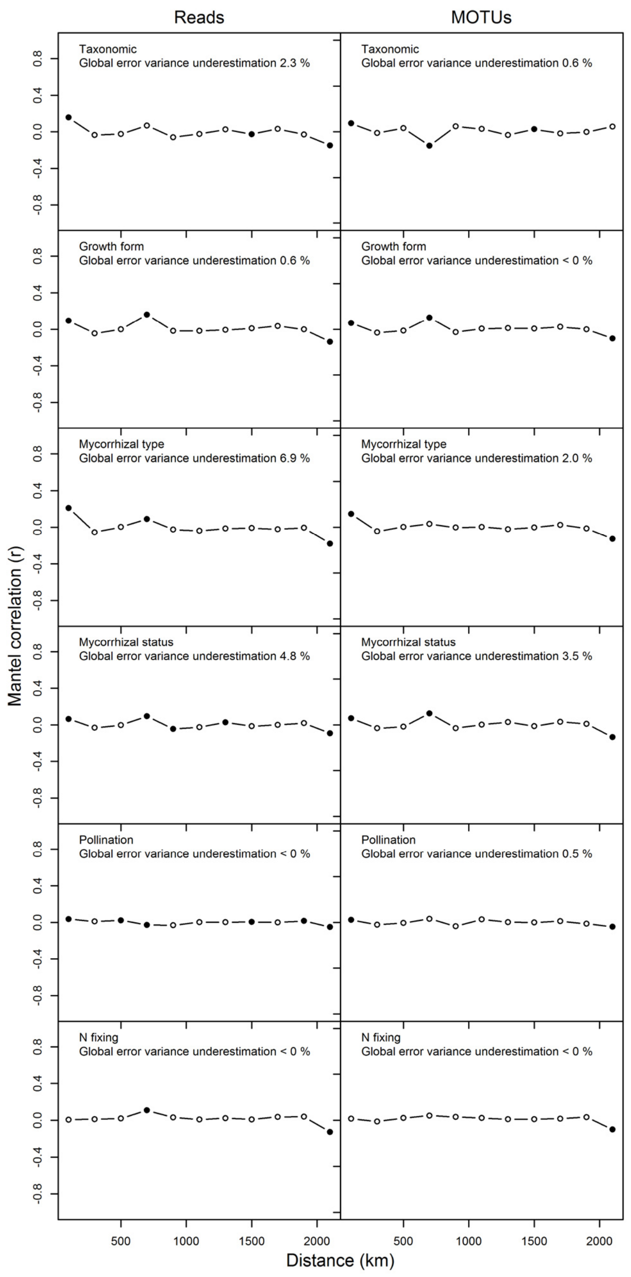

Supplementary Figure 1. Mantel correlograms depicting spatial autocorrelation in multivariate models of taxonomic or trait composition in relation to climatic period (pre-LGM, LGM, post-LGM). The residuals from distance-based redundancy analysis (db-RDA) models were used to estimate correlation at different spatial scales (with a spatial bin of 200 km). Models with proportional read composition (left-hand column) and proportional taxon composition (or presence-absence in the case of taxonomic composition; right-hand column) are presented separately. Significantly non-zero correlation was assessed using permutation (n=999) and is denoted with a filled symbol. Multi-scale ordination, a method based on canonical correspondence analysis[1], was used to estimate the degree to which observed autocorrelation leads to underestimation of the global error variance.

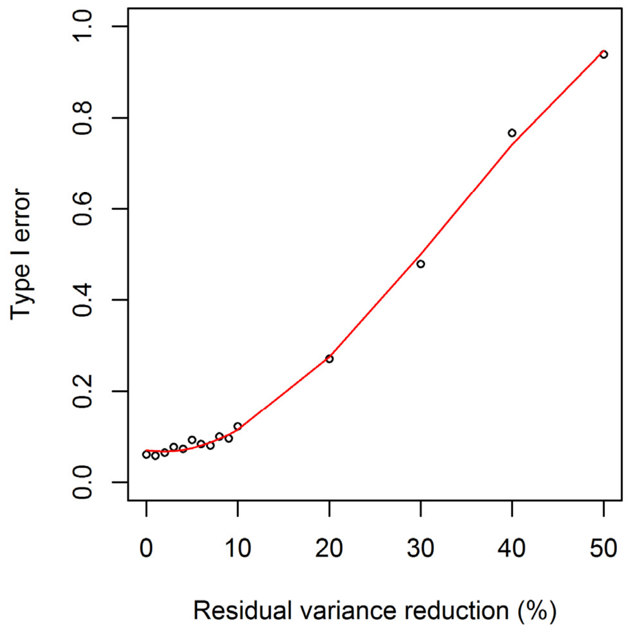

Supplementary Figure 2. Influence of residual variance underestimation on the Type I error rate in PERMANOVA. Type I error was estimated using permuted versions of the permafrost data set (taxonomic), such that no correlation with climatic period (pre-LGM, LGM, post-LGM) should be expected. The effect of climatic period on composition in 1000 randomly permuted data sets was calculated using PERMANOVA. The significance of the effect was tested in each model using further permutation (n=999). The proportion of significant results among the 1000 replicates should reflect the Type I error rate. The Type I error rate was calculated in this way for data sets where the residual variance was artificially reduced by between 1% and 50%. Type I error at residual variance reduction = 7% (i.e. the maximum degree of underestimation detected in this study) was approximately 8%, compared with the nominal rate of 5%. A cubic polynomial line of best fit is shown in red.

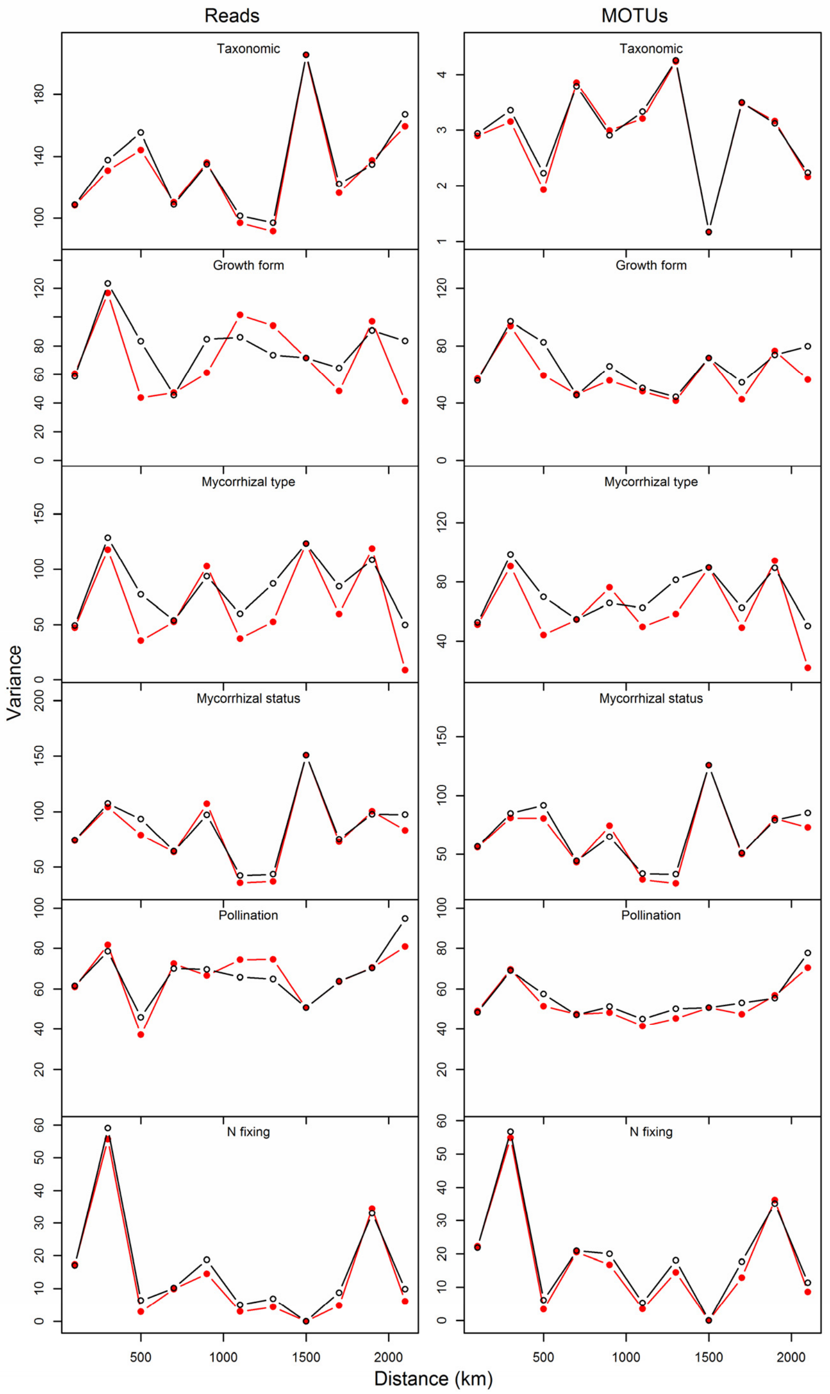

Supplementary Figure 3. Scale-dependence of species- and trait-climate relationships. Multi-scale ordination (a method based on canonical correspondence analysis[1]) of taxonomic or trait composition in relation to climatic period was carried out. Total variance (filled red points) and the sum of explained plus residual variance (open black points) are show for different spatial scales. A systematic difference between these lines can indicate a scale-dependent species-environment correlation.

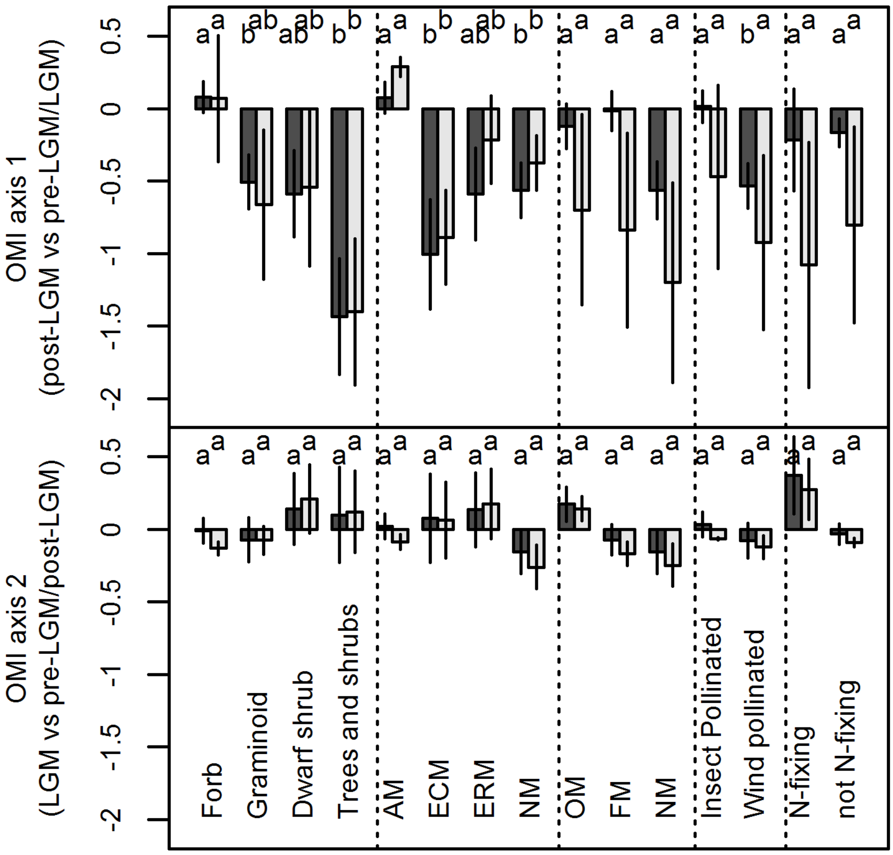

Supplementary Figure 4. Model estimates of mean OMI axis scores among plants belonging to different trait categories in phylogenetically-informed (dark grey) and non-phylogenetic analyses. Generalised least squares models with or without phylogenetic correction - in the form of Pagel's lambda correlation structure – were used to estimate model means. Error bars indicate model standard errors.

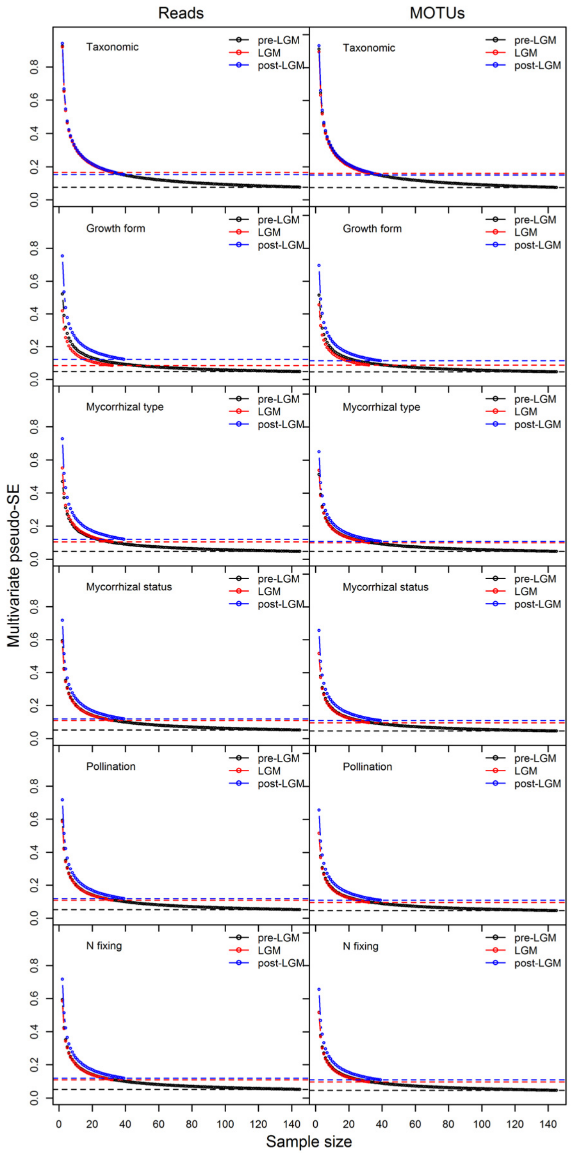

Supplementary Figure 5. The precision of multivariate estimates of taxonomic trait composition in Arctic permafrost samples. A distance-based analogue of the standard error (multivariate pseudo-SE;[2]) was calculated for trait composition within three different climatic periods (pre-LGM, LGM, post-LGM). This approach uses a double resampling procedure to describe variation in the position of the multivariate group centroid in relation to sampling effort. While the exact value of the parameter depends upon the dissimilarity measure used, the method allows users to assess the degree to which precision continues to improve with additional sampling and to compare between groups of samples. Here, we show that precision is already relatively high and similar for all groups, even the LGM and post-LGM, for which lower sample sizes were available. Dashed lines indicate the estimate of multivariate pseudo-SE for each group at the maximal sample size. Estimates are presented separately for estimates of composition based on read abundance (left hand column) and on MOTU abundance (right-hand column; presence-absence data in the case of taxonomic composition).

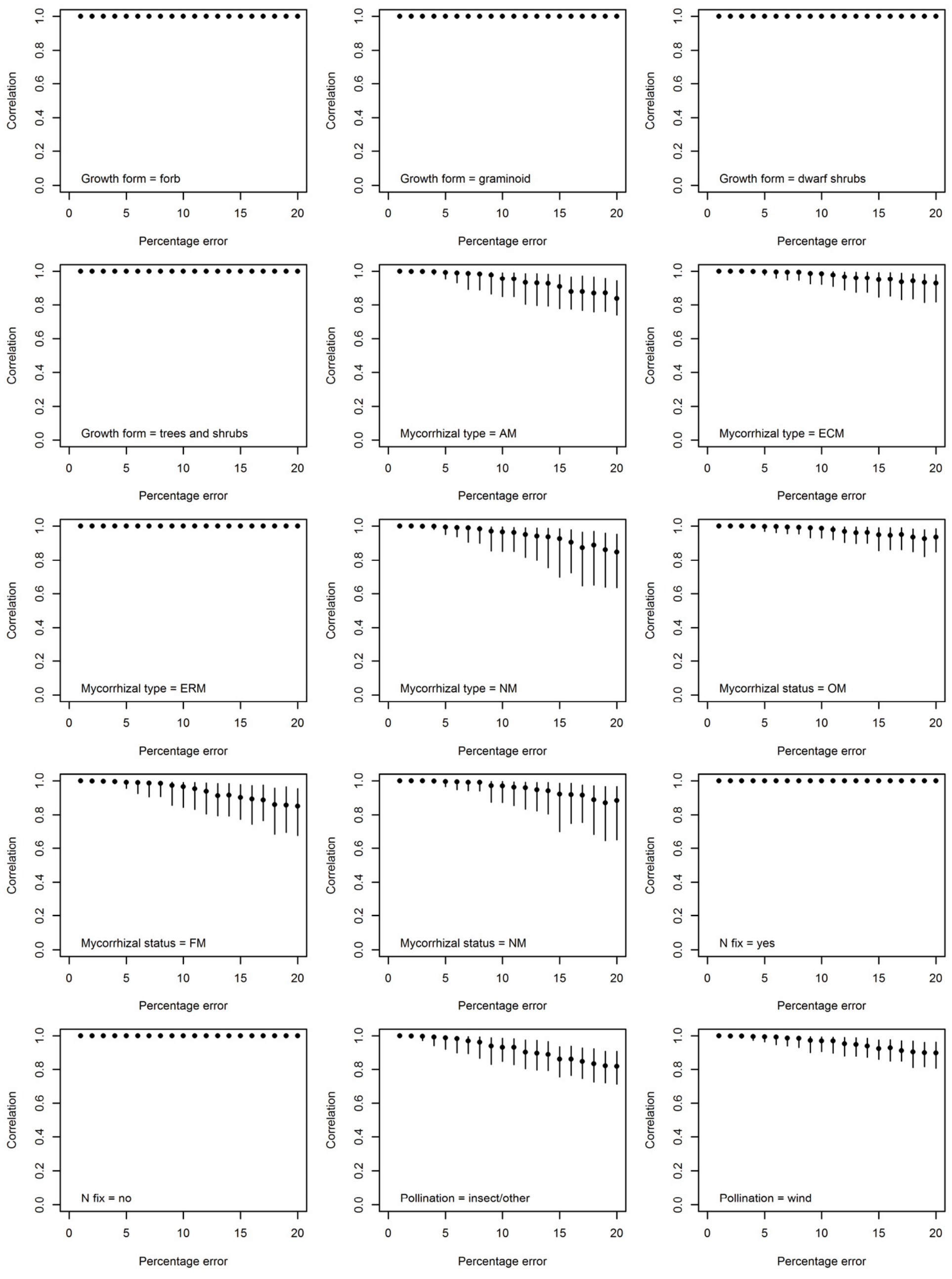

Supplementary Figure 6. Correlation (r) between per sample abundance of particular trait categories in the original data and data sets incorporating various levels of randomly-introduced error. The error rate was varied between 1% and 20% of species. Points indicate the median r, and bars indicate quartiles of r, resulting from 1000 random iterations. Note that growth form, N-fix and mycorrhizal type (ERM) were considered reliable trait categorisations and were not varied.

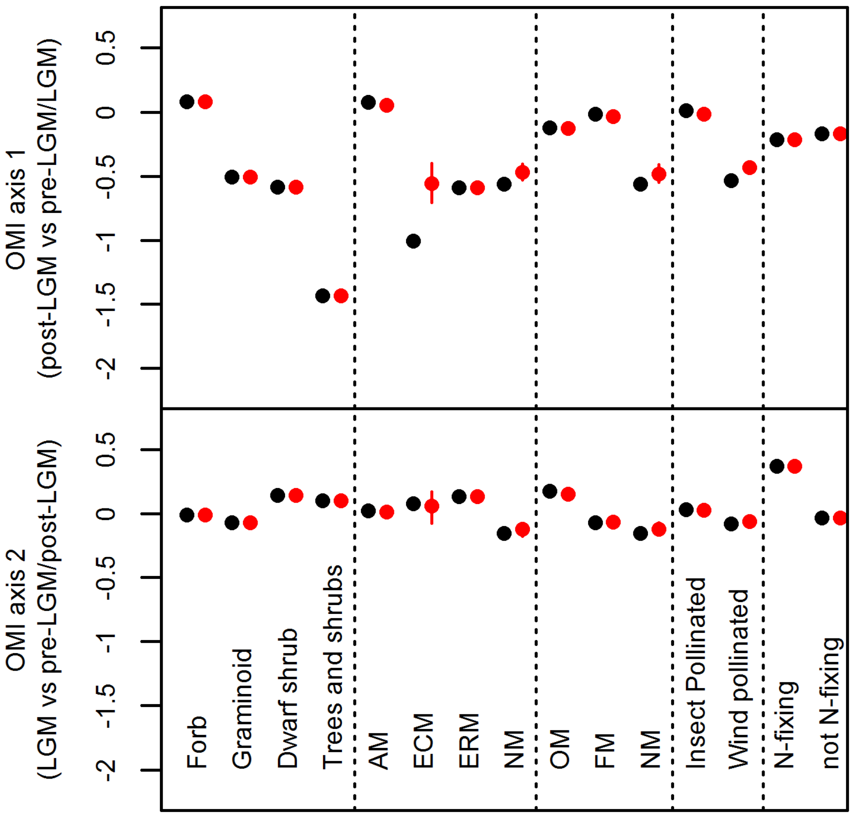

Supplementary Figure 7. Comparison of ordination (OMI) plant trait axis scores estimated from the original data (black) and data sets where randomly-introduced error was assigned to 10% of species (red). For the random error data, points indicate the median axis score, and bars the quartiles of axis scores, resulting from 1000 random iterations. See Fig. 3 for further interpretation.

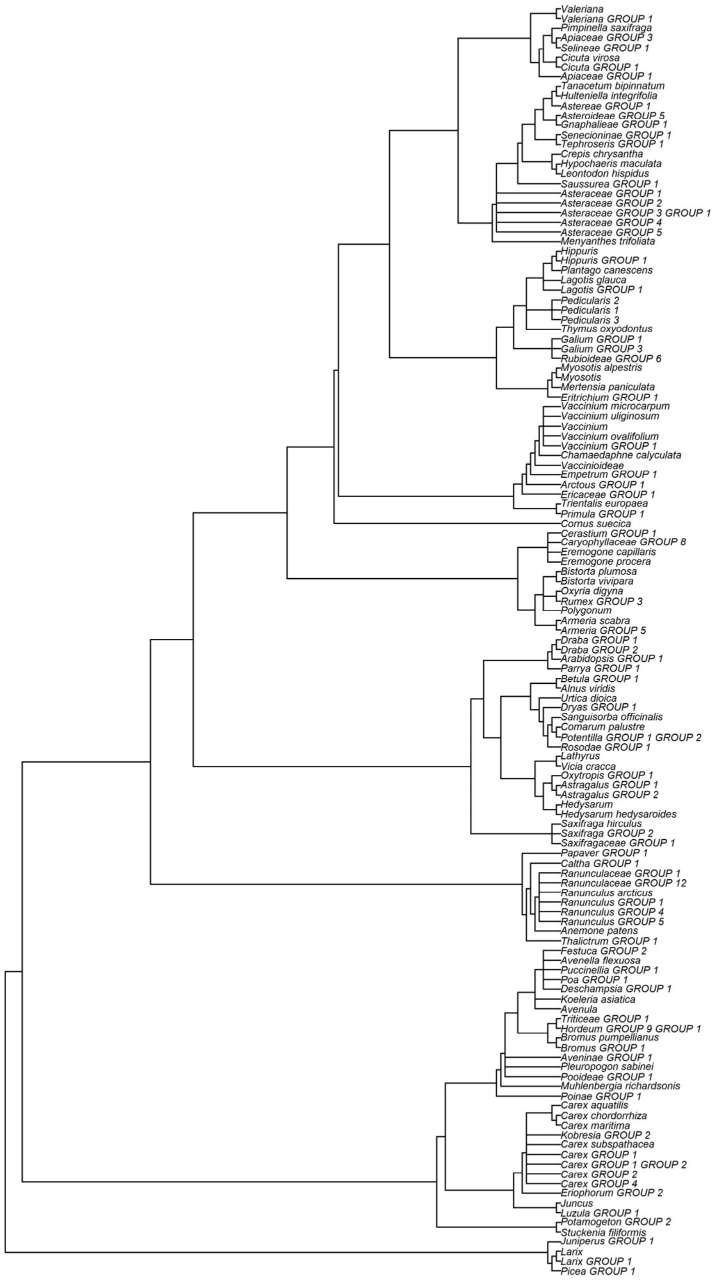

Supplementary Figure 8. Phylogenetic tree depicting the northern plant MOTUs from Willerslev et al.[3] used in this study. The phylogenetic placement of MOTUs was estimated by grafting tips onto a backbone supertree[4] at the lowest node positions that contained all MOTU component taxa (see Methods for details).

Supplementary Table 1. P values from PERMANOVA models of trait composition in relation to climatic period (pre-LGM, LGM, post-LGM) adjusted to account for variance underestimation associated with spatial autocorrelation (N = 216). The models were analogous to those described in Table 2, except that these were calculated using pseudo-F values derived from an artificially inflated estimate of residual variance. Residual variance was increased to the degree indicated by the respective MSO analysis (see Supplementary Fig. 1). The P values from this analysis are extremely similar to those in Table 2.

Trait	Read abundance	Taxon abundance
	Age	Age
Growth form	P < 0.001	P = 0.05
Mycorrhizal type	P < 0.001	P = 0.004
Mycorrhizal status	P = 0.01	P = 0.05
Pollination	P = 0.07	P = 0.24
N fixing	P = 0.04	P = 0.05

Supplementary References

1. Wagner, H. H. Direct multi-scale ordination with canonical correspondence analysis. *Ecology* **85**, 342-351 (2004).

2. Anderson, M. J. & Santana-Garcon, J. Measures of precision for dissimilarity-based multivariate analysis of ecological communities. *Ecology Letters* **18**, 66-73 (2015).

3. Willerslev, E., Davison, J., Moora, M., Zobel, M., Coissac, E., Edwards, M. E., Lorenzen, E. D., Vestergard, M., Gussarova, G., Haile, J., Craine, J., Gielly, L., Boessenkool, S., Epp, L. S., Pearman, P. B., Cheddadi, R., Murray, D., Brathen, K. A., Yoccoz, N., Binney, H., Cruaud, C., Wincker, P., Goslar, T., Alsos, I. G., Bellemain, E., Brysting, A. K., Elven, R., Sønstebø, J. H., Murton, J., Sher, A., Rasmussen, M., Ronn, R., Mourier, T., Cooper, A., Austin, J., Moller, P., Froese, D., Zazula, G., Pompanon, F., Rioux, D., Niderkorn, V., Tikhonov, A., Savvinov, G., Roberts, R. G., Macphee, R. D. E., Gilbert, M. T. P., Kjaer, K. H., Orlando, L., Brochmann, C. & Taberlet, P. Fifty thousand years of Arctic vegetation and megafaunal diet. *Nature* **506**, 47-51 (2014).

4. Durka, W., & Michalski, S. G. Daphne: a dated phylogeny of a large European flora for phylogenetically informed ecological analyses. *Ecology* **93**, 2297-2297 (2012).
